# What do employees on long-term sick leave experience, as barriers for returning to work?

**DOI:** 10.1093/eurpub/ckac129.316

**Published:** 2022-10-25

**Authors:** K Strømstad, LS Skarpaas, SE Wiik, SI Haslerud, RW Aas

**Affiliations:** 1 Department of Public Health, University of Stavanger, Stavanger, Norway; 2 Departmentof Occupational Therapy, Prosthetics and Orthotics, OsloMet – Oslo Metropolitan University, Oslo, Norway

## Abstract

**Background:**

The financial burden long-term sick leave places on society are immense and amounted to an annual cost of 180 billion NOK in Norway. Epidemiological and sociodemographic risk factors related to sickness absence and return to work (RTW) are well studied, less is known regarding self-perceived biopsychosocial barriers for RTW. The aim of this study was to investigate the diversity of barriers for RTW as experienced by long term sick listed employees.

**Methods:**

The study is a large-scale qualitative interview study (n = 85), using semi-structured telephone interviews. Participants were eligible to participate if they had received sick leave benefits >6 months and <1,5 years at the time of recruitment, for at least 50% of their employed work hours. The data was analysed with a directed qualitative content analysis combined with a summative approach. A deductive approach, guided by the theoretical framework provided in Model of Human Occupation (MoHO) were used in the analysis process. In MoHO, the main categories are person specific components and environmental components.

**Results:**

The study generated 952 coded meaning units describing barriers for RTW. Of these, we were able to deductively code 917 within the framework of MoHO. In the person specific concept, performance capacity barriers were dominant (n = 530). Volitional barriers (n = 164) were related to personal causation, hereunder self-efficacy (n = 24), and one's sense of capacity (n = 91). Barriers related to habituation (n = 64) was expressed as habits, both necessary habits and undesirable habits. Barriers related to the environmental component amounted to 388. The majority was linked to occupational environment (n = 217), including availability of adequate work tasks and barriers related to the healthcare system.

**Conclusions:**

The experienced RTW-barriers extended beyond health-related barriers, for most of the participants the barriers were related to both person specific components and environmental components.

**Key messages:**

• By gaining a greater understanding of the experienced RTW-barriers we could possibly provide more tailored RTW-services and help sick listed to a safe and sustainable return to work.

• The experienced RTW barriers for long term sick listed were primarily related to person specific and environmental components, and thus extended beyond health-related barriers.

